# Supplementary material for: Non-synonymous to synonymous substitutions suggest that orthologs tend to keep their functions, while paralogs are a source of functional novelty
Source: PeerJ. 2022 Aug 31;10:e13843. doi: 10.7717/peerj.13843 (PMC9440661; doi:10.7717/peerj.13843)
Supplement: Supplemental Information 1 [file peerj-10-13843-s001.pdf]

Supplementary figures

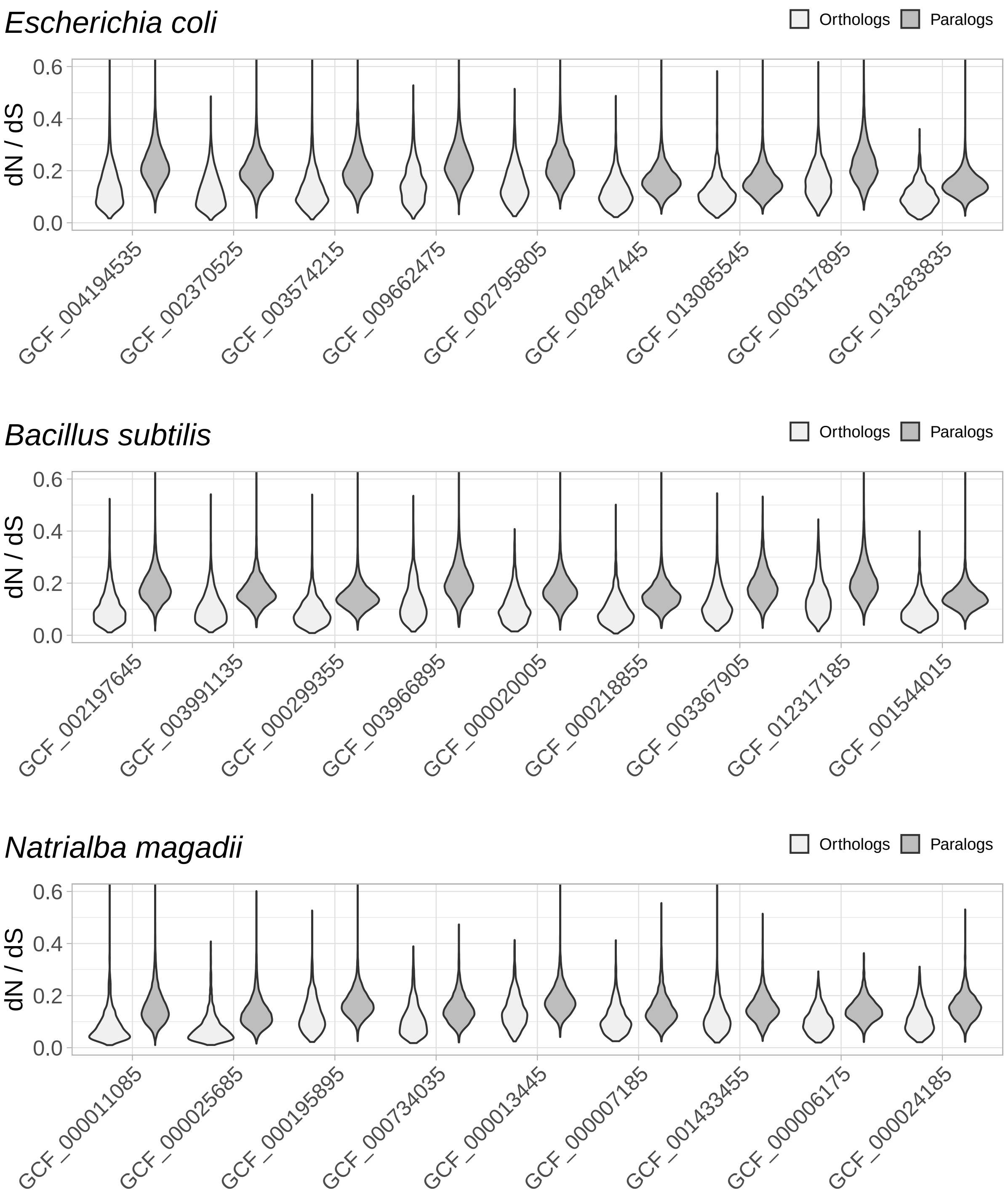

Figure S1: Full dN/dS results obtained with InParanoid as a working definition of orthology.

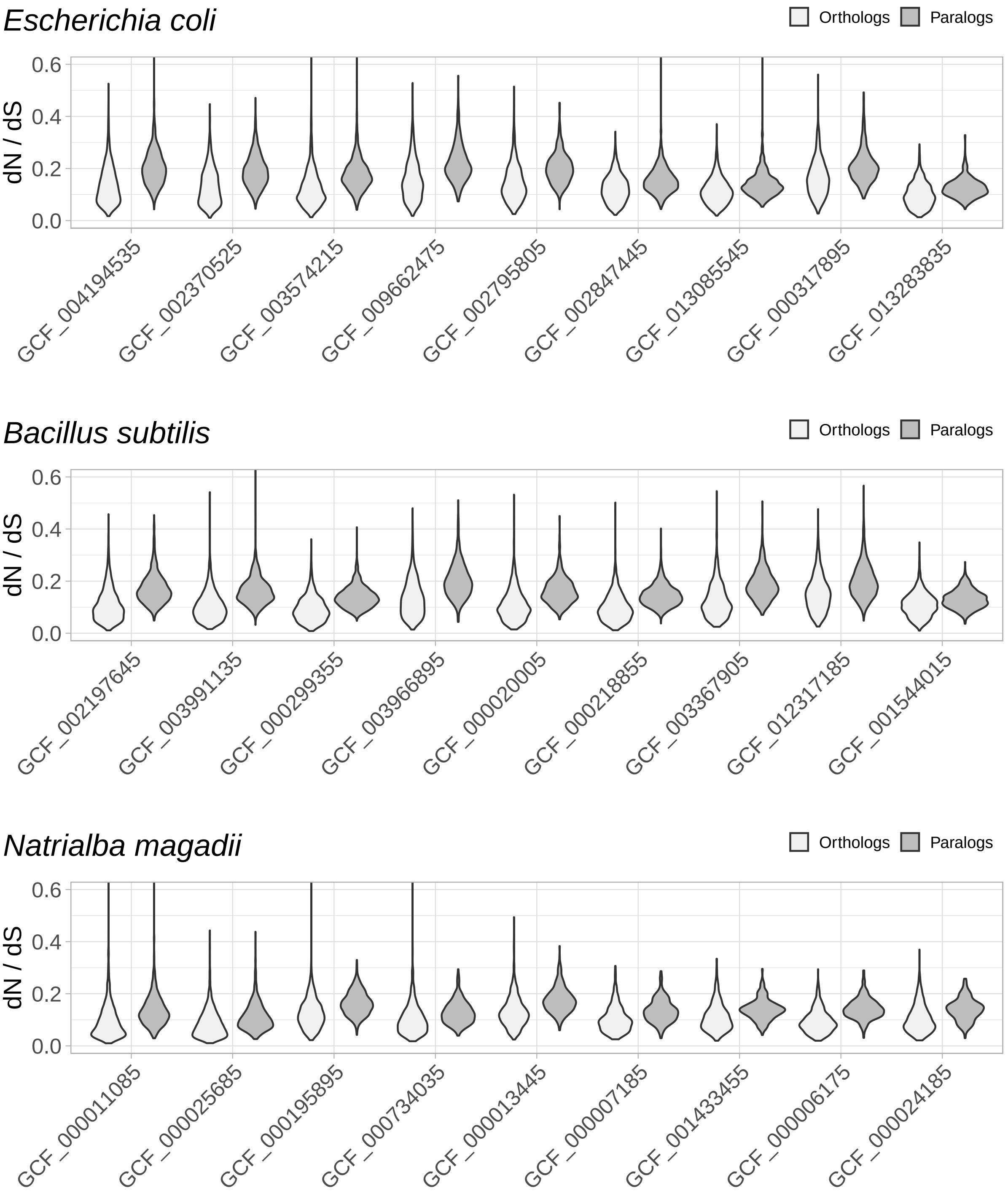

Figure S2: Full dN/dS results obtained with the Orthologous Matrix (OMA) working definition of orthology.

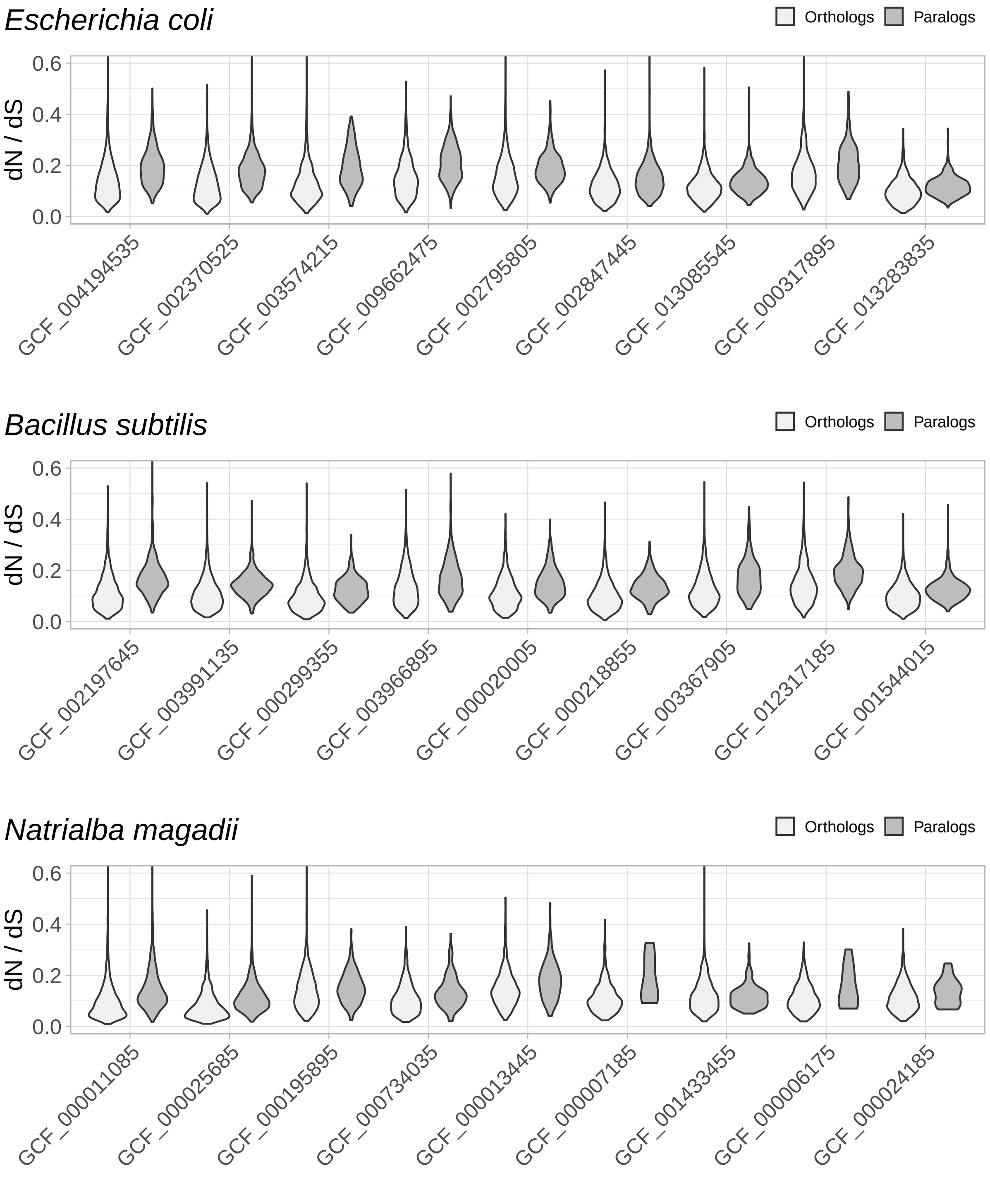

Figure S3: Full dN/dS results obtained with the OrthoFinder working definition of orthology.

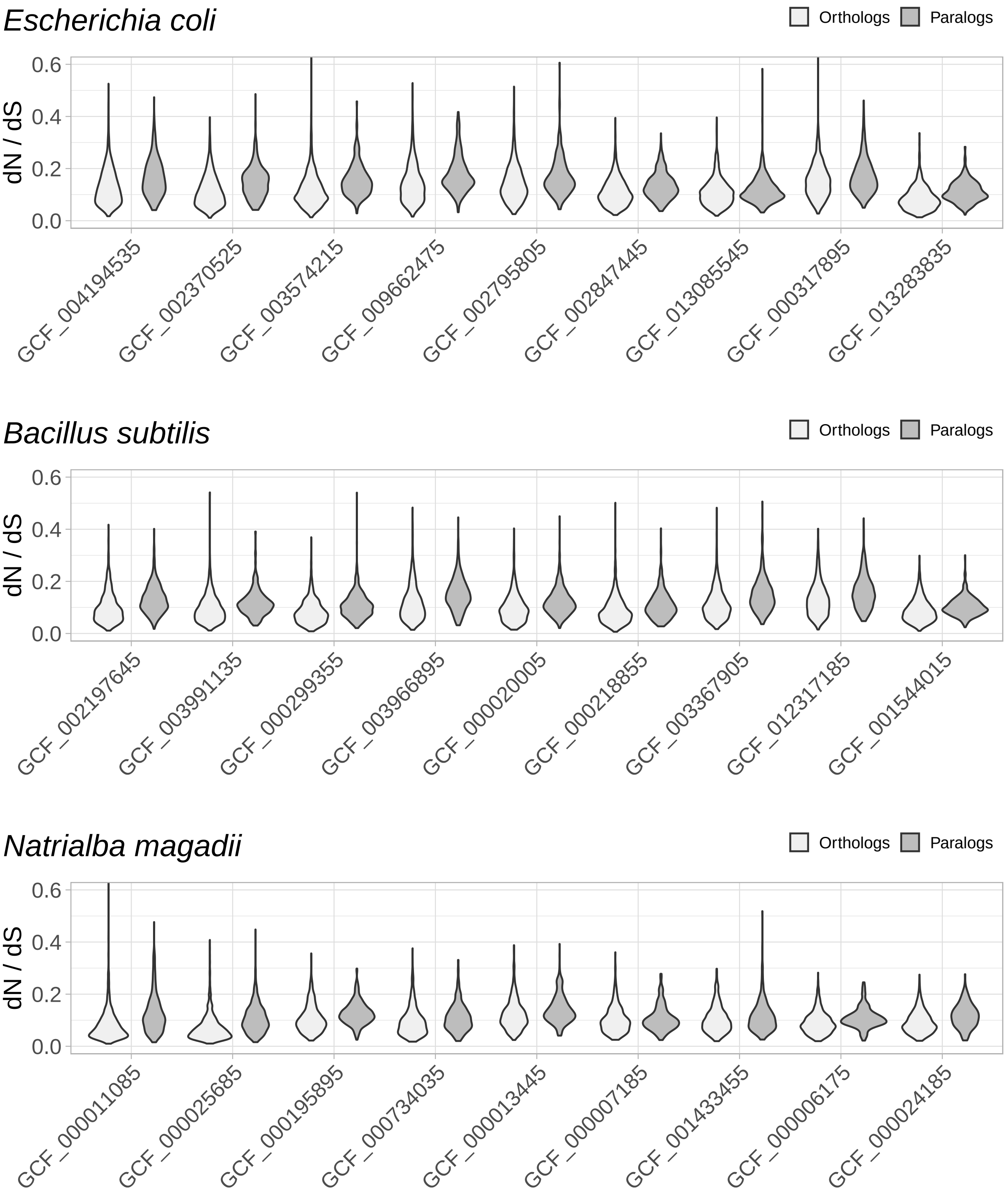

Figure S4: Full dN/dS results obtained with the ProteinOrtho working definition of orthology.

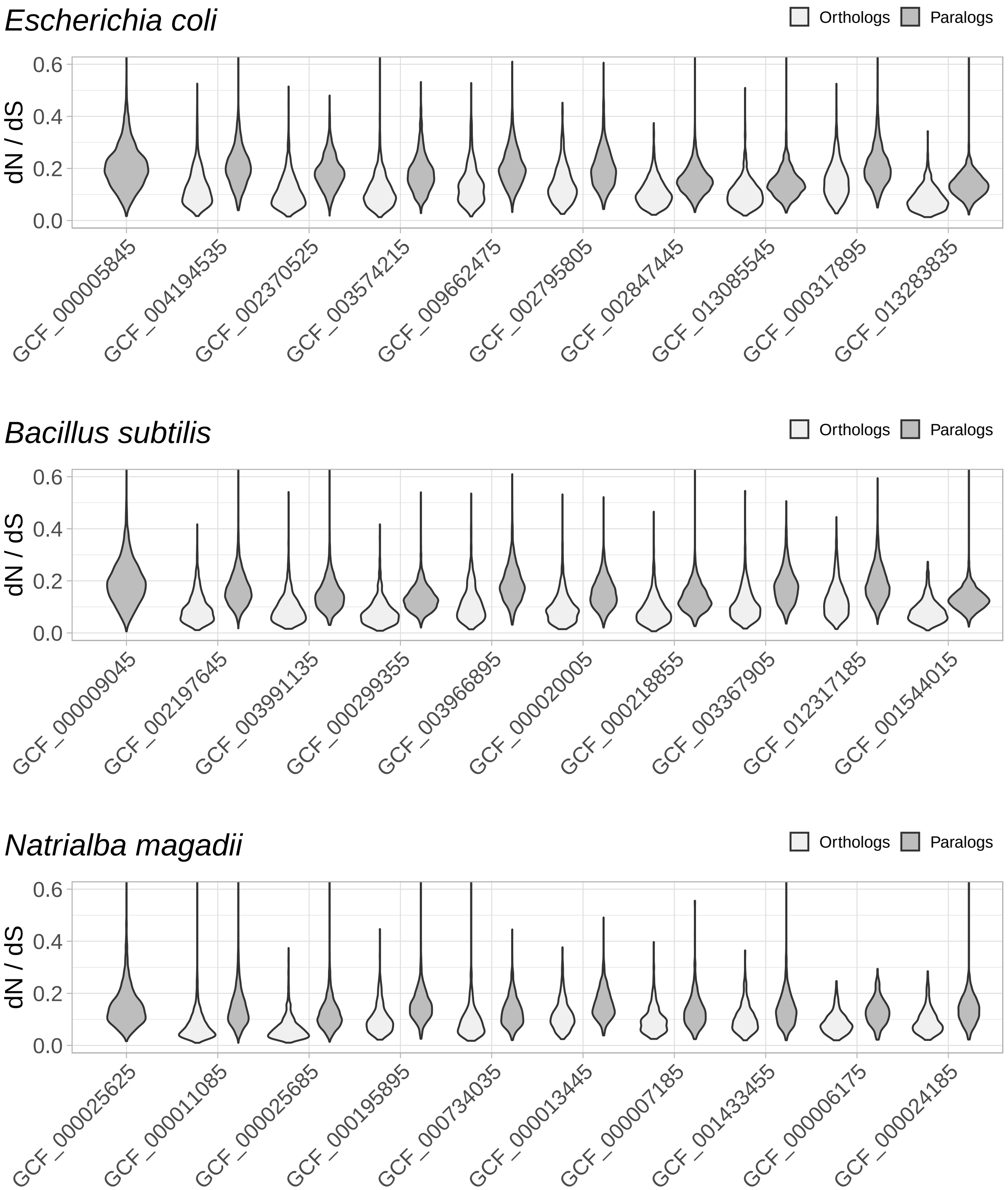

Figure S5: Full dN/dS results obtained with alignments covering at least 80% of both proteins.

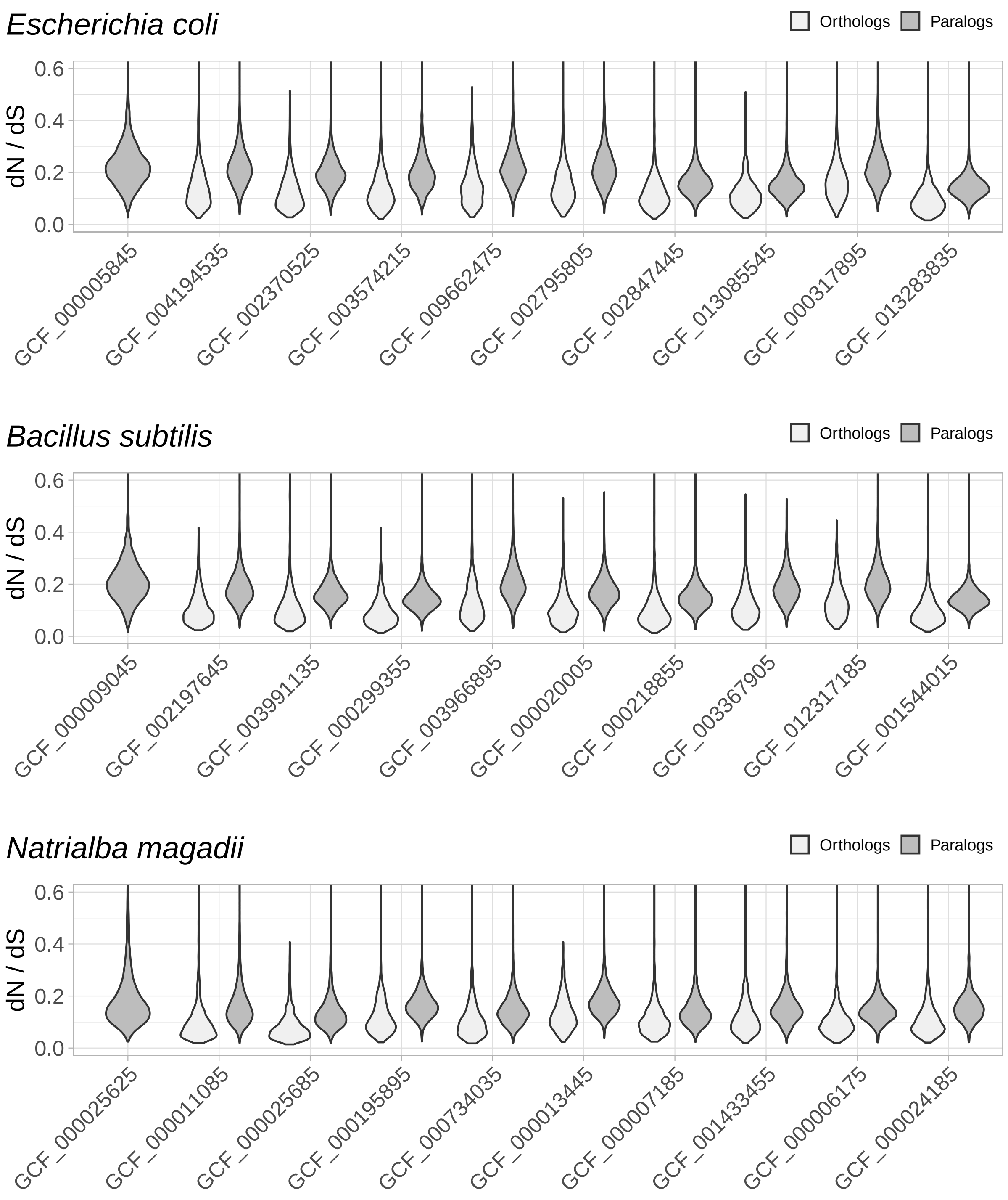

Figure S6: Full dN/dS results obtained with proteins no more that 70% identical.

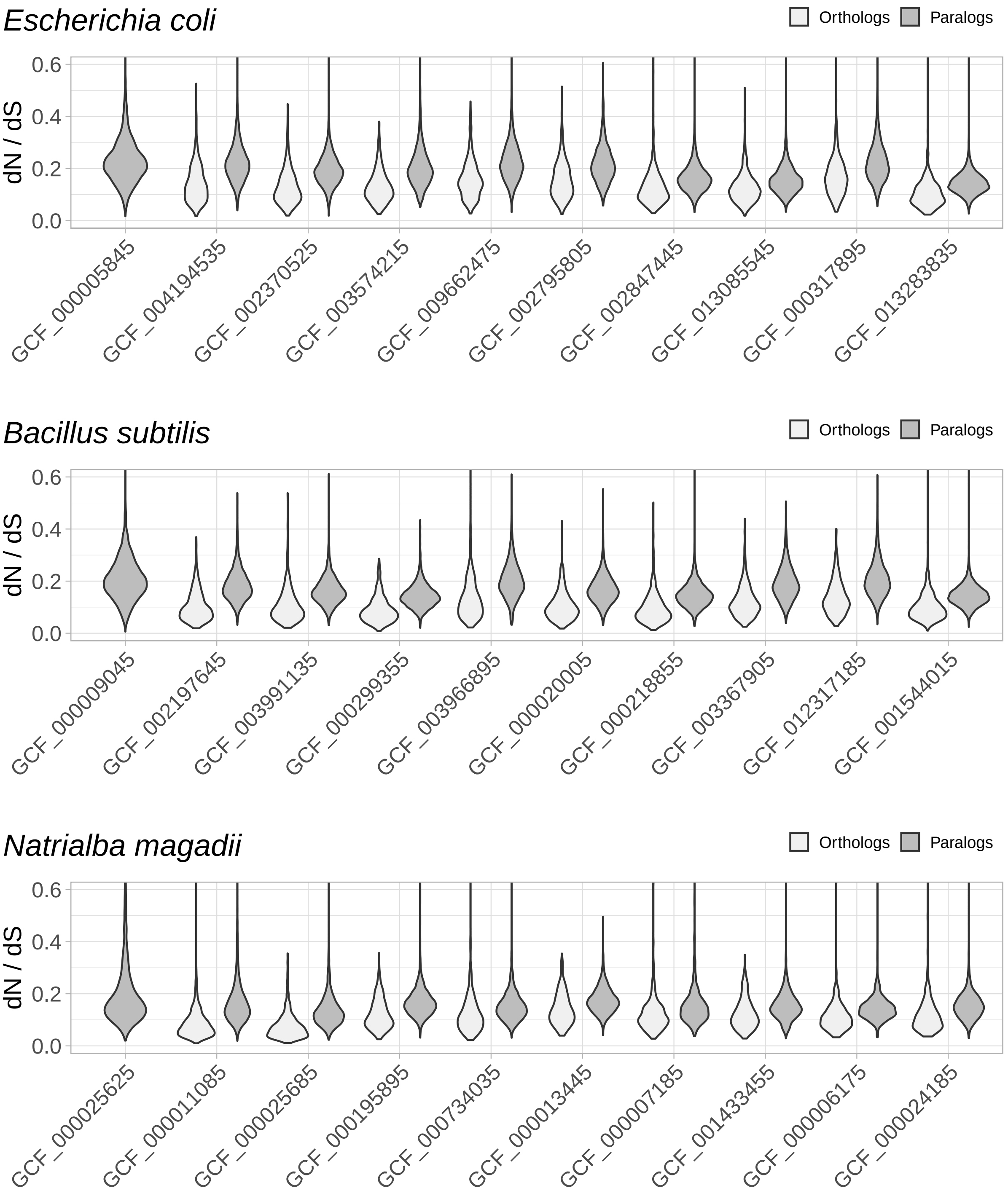

Figure S7: Full dN/dS results obtained with proteins within usual codon usage.

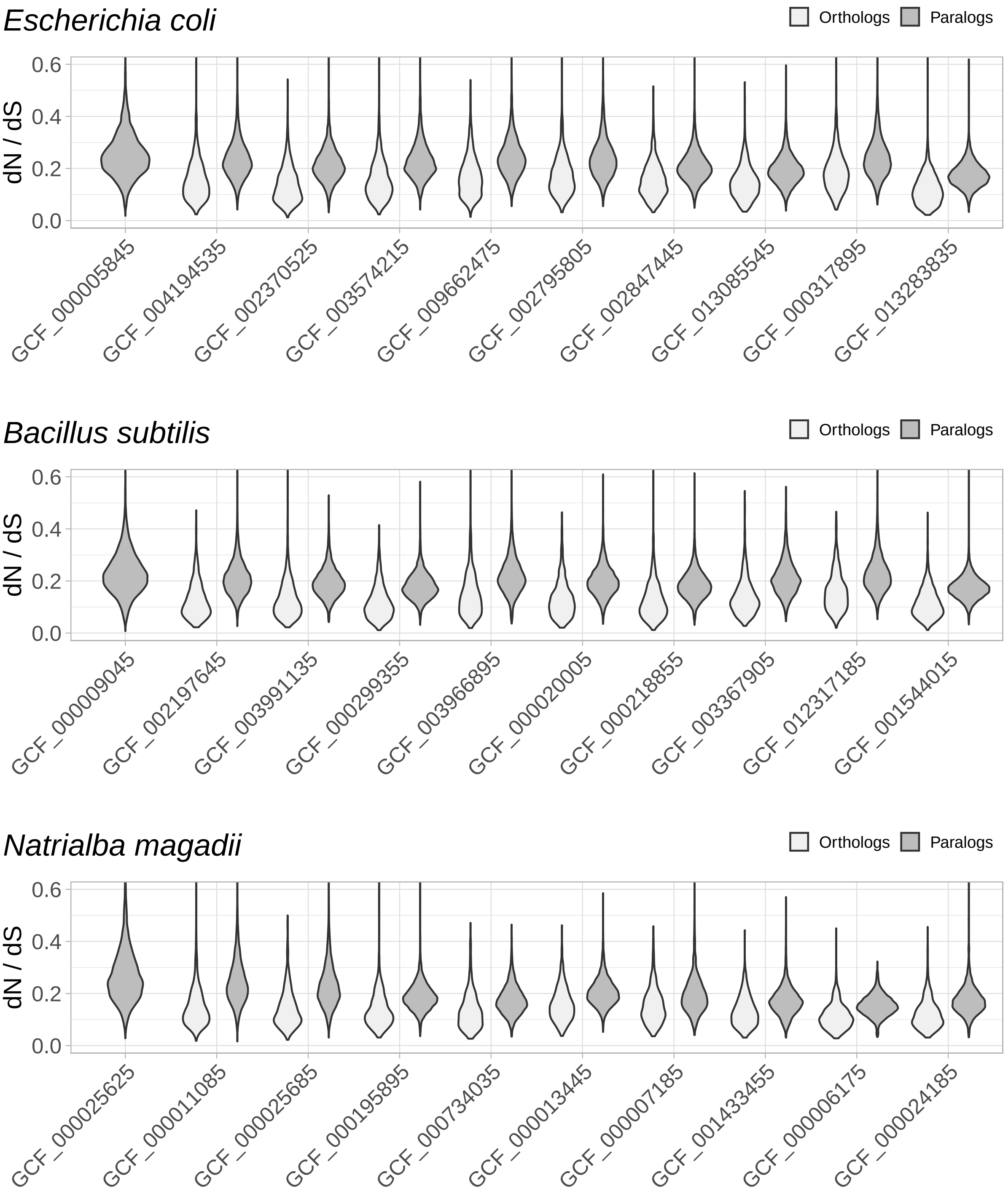

Figure S8: Full dN/dS results using Muse and Gaut's estimate of background codon frequencies.
